# Prevalence and predictors of over-the-counter medication use among pregnant women: a cross-sectional study in the Netherlands

**DOI:** 10.1186/1471-2458-13-185

**Published:** 2013-03-02

**Authors:** Gwenny MPJ Verstappen, Elise J Smolders, Janna M Munster, Jan G Aarnoudse, Eelko Hak

**Affiliations:** 1 University of Groningen, University Center for Pharmacy, PharmacoEpidemiology&PharmacoEconomics, Antonius Deusinglaan 1, 9713 AV, Groningen, The Netherlands; 2 Department of Obstetrics and Gynecology, University of Groningen, University Medical Center Groningen, Hanzeplein 1, Groningen, 9713GZ, The Netherlands

**Keywords:** OTC-medication, Pregnancy, The Netherlands, Epidemiology, Prediction model

## Abstract

**Background:**

Over-the-counter-medication (OTC-medication) use during pregnancy can be potentially harmful for the fetus. To successfully counsel the patient it is important to know if the patient is at risk. In this study possible predictors for OTC-medication use were identified and a model was designed to predict OTC-medication use during pregnancy.

**Methods:**

We performed a post-hoc analysis on data collected for a clustered clinical trial to study a screening strategy for Query fever. Pregnant women under supervision of a midwife were eligible for inclusion. These women filled out questionnaires during their pregnancy and post-partum. These questionnaires were used to determine the prevalence and to select possible predictors for OTC-medication use. These predictors were included in a prediction model using multivariate analysis. The discrimination and calibration of the model were assessed with Receiver Operating Characteristic analysis and the Hosmer and Lemeshow test.

**Results:**

Of the 1348 women enrolling in the clustered clinical trial, we included 1246 women in this analysis. The prevalence of OTC-medication use was 12.5%. The predictors for OTC-medication use in our cohort were: nulliparity, use of prescription medication, the presence of a comorbidity, Body Mass Index between 26 and 30 kg/m^2^ and General Practitioner visits. These predictors were used to design a prediction model for OTC-medication use. The area under the Receiver Operating Characteristic-curve of the prediction model was 0.667 (95% CI 0.620-0.714 P<0.001) and the predictive probabilities ranged from 6.6% to 57.4%. The Hosmer and Lemeshow goodness-of-fit test indicated good calibration of the model (P = 0.640).

**Conclusion:**

It is possible to indicate women at risk for OTC-medication use during pregnancy, using five maternal characteristics that independently contribute to the prediction model. The predictors are easy to estimate and the model is easy to implement in daily practice.

## Background

During pregnancy, there are some common ailments many women experience. Over-the-counter medications (OTC-medications) are widely used to treat these ailments. Because of their wide use, some OTC-medications (e.g. acetaminophen) are considered to be safe for pregnant women. However, by the absence of randomized clinical trials, the knowledge about the safety of OTC-medication exposure during pregnancy is dependent on observational studies. The only OTC-medications that are well-known to be potentially harmful are Non-Steroidal Anti-Inflammatory Drugs (NSAIDs). Fetal NSAID exposure is associated with an increased risk of constriction of the ductus arteriosus (DA) [1] and the risk of spontaneous abortion [2]. It is particularly difficult to investigate the consequences of OTC-medication use on fetal outcomes, because their use is not fully documented. Even healthcare providers are not always aware of OTC-medication use by their patient during pregnancy. In the Dutch self-care guidelines for healthcare professionals [3] most OTC-medications are discouraged to use during pregnancy because of the lack of evidence for either safety or fetal risk. Therefore, healthcare providers should provide personalized counseling to pregnant women about possible negative side effects of OTC-medication on their unborn child. In order to achieve this it is preferable to indicate women that are at risk for OTC-medication use.

The study results published so far on OTC-medication use during pregnancy are difficult to compare, because research populations and study designs differ strongly. For example, studies from the United States found percentages of women that self-medicated with OTC medication varying between 92,6% [4] and 53,6% [5]. Furthermore, drug utilization patterns vary strongly between different countries. It is therefore important to study the prevalence and predictors of OTC-medication use in a Dutch setting. Previous studies already investigated drug use during pregnancy in the Netherlands, but they used different study designs and did not focus on OTC-medication [6,7]. By describing possible predictors of OTC-medication use during pregnancy, midwifes and obstetricians in the Netherlands can be assisted in identifying pregnant women that are likely to use OTC-medication. In this population ‘at-risk’, personalized counseling can be given in order to mitigate medication-related risks for the unborn child. We have implemented the predictors in a prediction model applicable in daily practice.

## Methods

### Setting and participants

The dataset used in this cross-sectional study was primarily assembled for a clustered randomized controlled trial to study a screening strategy for Query fever (Q fever) among pregnant women. This clinical trial has been approved by the ethics committee of the University Medical Center Groningen [8]. Participants were recruited by midwife centers in Q fever affected areas in the Netherlands (southern part of the Netherlands; North Brabant). The study population comprised pregnant women under supervision of a midwife in primary healthcare. In the Netherlands, midwifes are only allowed to supervise low-risk, singleton pregnancies. Women were eligible for inclusion if they were 18 years of age or older, and pregnant with an estimated date of delivery between June 1^st^ and December 31^st^ 2010. Women returned the questionnaire by internet, by logging in to a protected website. Women were ineligible for inclusion if they had no access to internet/email, if they were unable to understand Dutch or unable to give informed consent.

### Data collection

We used data from two different questionnaires. The first questionnaire was filled out in the second or third trimester of pregnancy and included questions about the current pregnancy, outcome of previous pregnancies, lifestyle, comorbidities not related to pregnancy (e.g. cardiac disease, lung disease), (OTC-)medication use during pregnancy and socio-demographic characteristics. The second questionnaire was completed by the participants one month post-partum. With this questionnaire health complaints and healthcare use during and one month after pregnancy were examined. Data from both questionnaires were used to study possible predictors for OTC-medication use during pregnancy.

We categorized all medication that is available without a prescription from a doctor in the Netherlands as OTC-medication. All drugs were classified according to the Anatomical Therapeutic Chemical (ATC) classification system of the World Health Organization [9] and divided in subgroups based on complaints for which the drugs were used. In case of small groups, different types of medication were clustered in one group.

Folic acid and vitamin D are vitamins which are advised in the Netherlands during pregnancy [10]. Therefore, women who have only used these vitamins are categorized as non-OTC-medication users. When prenatal vitamins were reported, we considered them as multivitamins designed for pregnant women containing more and/or other vitamins than folic acid and vitamin D. Because prenatal vitamins are not actively advised by midwifes in the Netherlands during pregnancy, we considered them as OTC-medication.

### Outcome measures and predictor selection

The primary endpoint, the prevalence of OTC-medication use, was calculated by using descriptive statistics. We evaluated whether the primary endpoint could be predicted by maternal characteristics. Therefore, a prediction model was developed. For each candidate predictor included in this model at least ten events were required [11]. Candidate predictors are listed in Table 1. We only selected predictors that were available in our dataset.

**Table 1:** Candidate predictors of OTC-medication use during pregnancy

**Socio-demographic predictors**	Education level, age, ethnicity
**Comorbidity predictors**	Use of prescription drugs, chronic illness, healthcare consumption, health complaints during pregnancy
**Lifestyle predictors**	Smoking and alcohol use during pregnancy, Body Mass Index
**Obstetric history predictors**	Gravidity, parity, miscarriage, prematurity, child mortality

The possible maternal characteristics which can predict OTC-medication use during pregnancy. Marital status is not included among the socio-demographic predictors, because these data were not available.

### Statistics

The reported OTC­medications were divided into sub­categories based on the complaints for which the medications were used. Dermatics, homeopathy, comforting medication were combined into one category for statistical reasons. Some characteristics (i.e. Body Mass Index (BMI), ethnicity, education) were also divided into sub-categories. For BMI, a distinction was made between underweight (<19 kg/m^2^), ideal weight (19–25 kg/m^2^), overweight (26–30 kg/m^2^) and obesity (>30 kg/m^2^). However, for statistical reasons, we grouped underweight, ideal weight and obesity as one reference category. For ethnicity, the sub-category ‘Western’ includes women of Dutch origin and other Western origins. The sub-category ‘Non-western’ includes all other origins (e.g. African, Asian, Hindu). We divided education in a ‘low’, ‘middle’ and ‘high’ sub-category. High education includes higher vocational education, middle education includes intermediate vocational education and low education includes all education levels that are lower than intermediate vocational education. We performed univariate analysis for the single associations using Pearson’s Chi-square tests to calculate odds ratios (OR) and 95% Confidence Intervals (95% CI). For all the statistical analyses we excluded the missing values. After the univariate analysis, we included the candidate predictors in a multivariate analysis using logistic regression. We used backward elimination to exclude non-contributable predictors (P ≥0.05) from the prediction model. The Hosmer and Lemeshow goodness-of-fit test was used to measure how well the predicted probabilities by the model correspond to the observed probabilities. Furthermore, a calibration plot of the observed and predicted probabilities was made. Receiver Operating Characteristic (ROC) analysis was used to test the discriminating performance of our model. For the clinical daily practice an equation to estimate the likelihood of OTC-medication use for the individual woman was designed, using the rounded regression coefficients of the multivariate analysis. All the analyses described above were performed with PASW Statistics version 18.0 (SPSS inc. Chicago, Illinois, USA).

## Results

Out of 6860 women that were eligible for the Q fever study, 1348 women responded and were included in the prior dataset [8]. Participants that had not completed the first questionnaire during pregnancy and participants that did not fill out the question concerning OTC-medication use were excluded from all the analyses. 1246 participants were eligible for final inclusion in the analysis. Of these women, 157 women (12.5%) reported OTC-medication use during pregnancy, with an average of 1.19 OTC-medications per woman. The most commonly reported medications were analgesics, (prenatal) vitamins and medication for the gastro-intestinal tract (Table 2).

**Table 2:** Prevalence of OTC-medications used by pregnant women

**OTC-medication classification**	Frequency*	**Percent**	**Valid percent**	**Cumulative percent**
Dermatics, Homeopathy, Comforting medication±	24	12.8	12.8	12.8
Analgesics‡	51	27.3	27.3	40.1
Cold & flu medication	22	11.8	11.8	51.9
Prenatal vitamins and other vitamins†	50	26.7	26.7	78.6
Gastro-intestinal tract medication	40	21.4	21.4	100.0
Total	187	100	100	

The prevalence of OTC-medication use calculated for each group of OTC-medication.

* Women that reported the use of two or more OTC-medications are included two times or more in this prevalence calculation.

† Users of only Folic acid and/or vitamin D are excluded.

± Comforting medication includes medication for sleep disorders.

‡Analgetics includes NSAID’s and acetaminophen.

The baseline characteristics and the results of the univariate analyses of the predictors in relation to overall OTC-medication use during pregnancy are shown in Table 3. Women were more likely to use OTC-medication if they: used prescription drugs (OR 2.36 95% CI 1.54-3.62), were nulliparous (OR 1.66 95% CI 1.13-2.33), had their first pregnancy (OR 1.57 95% CI 1.13-2.01), had a BMI between 26–30 kg/m^2^ (OR 1.49 95% CI 1.00-2.21), had health complaints during pregnancy (OR 2.19 95% CI 1.55-3.10), reported more GP visits during pregnancy (2 times: OR 1.74 95% CI 1,04-2.93 and ≥ 3 times OR 2.34 95% CI 1.30-4.22), reported extra visits to the midwife (1 time extra: OR 1.70 95% CI 1.05-2.73 2 times extra: OR 2.10 95% CI 1.12-3.91) and if they had a comorbidity (OR 2.37 95% CI 1.90-4.35).

**Table 3:** Baseline characteristics and results of univariate analyses of the predictors in relation to OTC-medication use

**Predctor**	**N**	**OTC-medication use (n)**	**%**	**No OTC-medication use (n)**	**%**	**df**	**P (Chi2)**	**OR (95% CI)**
**Prescription drugs**								
*Yes*	148	34	23.0	114	77.0	1	0.000	2.36 (1.54-3.62)
*No*	1097	123	11.2	974	88.8			
**Age**								
*18-35*	1080	132	12.2	948	87.8	1	0.305	
*>35*	166	25	15.1	141	84.9			1.27 (0.80-2.02)
**Ethnicity**								
*Western*	*1217*	150	12.3	1067	87.7	1	0.102	
*Non-western*	26	6	23.1	20	79.9			2.13 (0.84-5.40)
**Nulliparity**								
*Yes*	561	88	15.7	473	84.3		0.003	1.66 (1.19-2.33)
*No*	685	69	10.1	616	89.9	1		
**Gravidity**								
*1*	482	76	15.8	406	84.2	1	0.007	1.57 (1.13-2.01)
*2 or more*	764	81	10.6	683	89.4			
**Parity≠**								
*0*	561	88	15.7	473	84.3	3	0.028	1.84 (1.06-3.18)
*1*	500	52	10.4	448	89.6			1.15 (0.65-2.04)
*2 or more*	185	17	9.2	168	90.8			
Adverse obstetric outcome in history*								
*No*	946	124	13.1	822	86.9	1	0.338	1.22 (0.81-1.84)
*Yes*	300	33	11.0	267	89.0			
**Miscarriage**								
*Yes*	270	29	10.7	241	89.3	1	0.298	
*No*	976	128	13.1	848	86.9			1.25 (0.82-1.92)
**Prematurity/mortality**								
*No*	1200	150	12.5	1050	87.5	1	0.586	
*Yes*	46	7	15.2	39	84.8			1.26 (0.55-2.86)
**Educationτ**								
*Low*	88	7	8.0	81	92.0	2	0.377	
*Middle*	412	55	13.3	357	86.7			1.78 (0.78-4.06)
*High*	744	95	12.8	649	87.2			1.69 (0.76-3.78)
**Body Mass Index(kg/m**^**2**^**)Ξ**								
<26 >30	1013	119	11.70	894	88.30	1	0.049	
26-30	230	38	16.5	192	83.5			1.49 (1.00-2.21)
**Smoking during pregnancy**								
*No*	1129	145	12.8	984	87.2	1	0.422	1.29 (0.69-2.40)
*Yes*	117	12	10.3	105	89.7			
**Alcohol consumption during pregnancy**								
*No*	1216	151	12.4	1065	87.6	1	0.185	
*Yes*	29	6	20.7	23	79.3			1.84 (0.74-4.59)
**Health complaints**								
*No*	779	74	9.5	705	90.5	1	0.000	
*Yes*	401	75	18.7	326	81.3			2.19 (1.55-3.10)
**General Practitioner visits±**								
*0*	695	74	10,6	621	89,4	3	0,013	
*1*	260	34	13,1	226	86,9			1.26 (0.82-1.95)
*2*	128	22	17.2	106	82.8			1.74 (1.04-2.93)
*3 or more*	78	17	21.8	61	78.2			2.34 (1.30-4.22)
**Extra midwife visits†**								
*0*	929	104	11.2	825	88.8	3	0.028	
*1*	142	25	17.6	117	82.4			1.70 (1.05-2.73)
*2*	67	14	20.9	53	79.1			2.10 (1.12-3.91)
*3 or more*	51	7	13.7	44	86.3			1.26 (0.55-2.88)
**Other healthcare providers**								
*0*	1067	131	12.3	936	87.7	3	0.274	
*1*	23	2	8.7	21	91.3			0.68 (0.16-2.94)
*2*	15	1	6.7	14	93.3			0.51 (0.07-3.91)
*3 or more*	85	16	18.8	69	81.2			1.66 (0.93-2.94)
**Comorbidity‡**								
*No*	1099	119	10.8	980	89.2	1	0.000	
Yes	147	38	25.9	109	74.1			2.87 (1.90-4.35)

n = number of women in the group.

The reference categories are dichotomous.

* Adverse obstetric outcome in history includes all cases of miscarriage, prematurity and mortality of the child in the obstetric history.

† Extra midwife visits are visits beside the regular consults by the midwife. No extra midwife visits is the reference category.

‡ Comorbidity includes any kind of comorbidity (e.g. cardiac disease, lung disease and other chronic diseases) reported by the pregnant women. These comorbidities exclude pregnancy complications.

± No extra GP visits is the reference category.

≠ Never gave birth (parity = 0) is the reference category.

T Low-education level is the reference category.

Ξ BMI is pre-pregnancy BMI.

The next step was a multivariate analysis including predictors selected from the univariate analysis and known from literature [4,5,12-14]. Because data were missing for some variables 89 women were excluded for the multivariate analysis; 1157 women were included. These results are shown in Table 4. The model designed with the multivariate analysis includes only the variables that independently predict OTC use. These variables are: Prescription drug use, nulliparity, comorbidity, BMI 26–30 kg/m^2^ and the number of GP visits during pregnancy (1, 2, 3 or more than 3 times).

**Table 4:** Results of the multivariate analysis of the predictors in relation to OTC-medication use during pregnancy

**Predictor**	**B (regression coefficient)**	**Significance**	OR (95%CI)*
Prescription drugs†	0.418	0.101	1.52 (0.92-2.50)
Nulliparity‡	0.472	0.009	1.60 (1.12-2.29)
Comorbidity±	0.887	0.000	2.43 (1.53-3.87)
Body Mass Index (kg/m^2^) ≠	0.483	0.022	1.62 (1.07-2.46)
1 General Practitioner visit T	0.224	0.321	1.25 (0.80-1.95)
2 General Practitioner visits T	0.507	0.063	1.66 (0.97-2.83)
≥3 General Practitioner visits T	0.684	0.028	1.98 (1.07-3.65)

*Odds Ratio and the 95% confidential intervals.

† No use of prescription drugs is the reference category.

‡ Never been pregnant (nulliparity) is the reference category.

± No comorbidities is the reference category.

≠A BMI of <26 >30 kg/m^2 ^is the reference category.

T No extra visits to the GP is the reference category.

**Figure 1:**
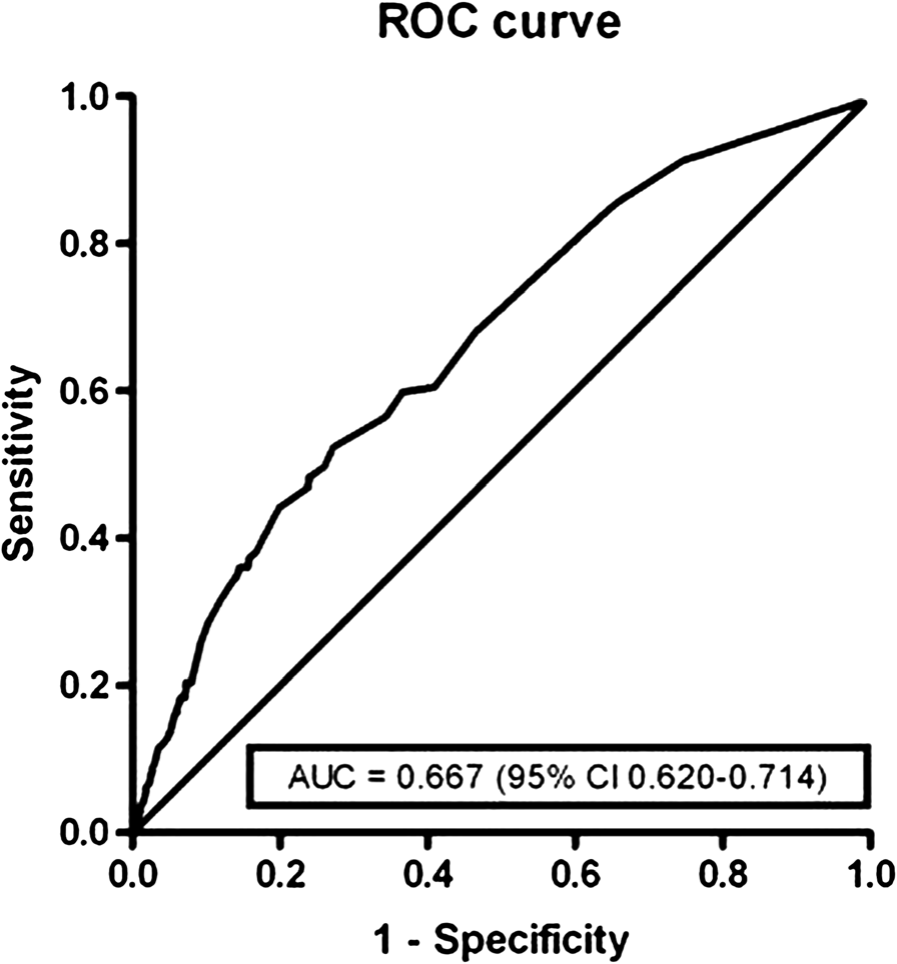
**ROC-curve of the multivariate analyses. **ROC-curve = Receiver-operating characteristic. AUC = area under the curve**. **95% CI = 95% confidential interval.

**Figure 2:**
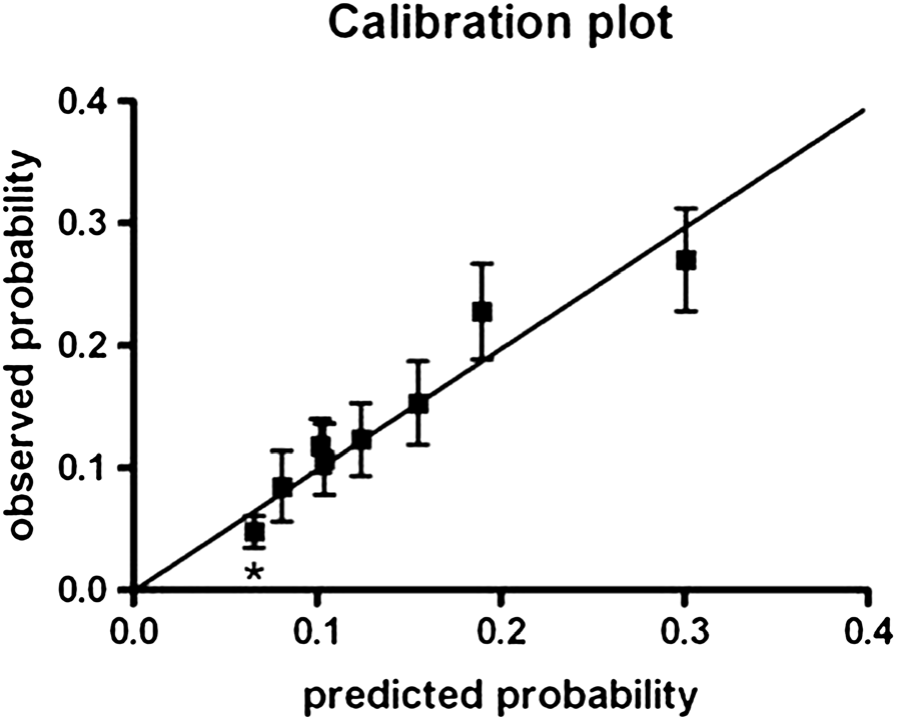
Calibration plot of the predicted probability (deciles) versus the observed probability. * Decile 1 till 3 were taken together since the predicted probabilities were equal.

The discrimination ability of the model was indicated by an area under the ROC-curve (Figure 1) of 0.667 (95% CI 0.620-0.714 P<0.001). For the individuals the predicted probability ranged from 6.6% to 57.4%. For the two highest groups of the predictive probabilities there is a small under- and overestimation of OTC-medication use (Figure 2). The P-value of 0.640 produced by the Hosmer and Lemeshow goodness-of-fit test supports that the model statistically fitted (P>0.05). For the clinical daily practice an equation to calculate individual risk scores was designed (Equation 1). This score is an indication for the probability that a pregnant woman will use OTC-medication. The maximum score is 13, indicating the highest chance of OTC-medication use, and the minimum score is 0, indicating the lowest chance of OTC-medication use.

### Equation 1: Risk score for the probability of OTC-medication use during pregnancy

$$\begin{array}{l} \text{Risk}\;\text{score}=2\;\times \;\text{prescription}\;\text{drug}+2\;\\ \;\times \;\text{nulliparity}+4\;\times \;\text{comorbidity}+2\;\\ \;\times \;\text{BMI}+\text{number}\;\mathrm{of}\;\text{visits}\;\mathrm{GP} \end{array}$$

Prescription drug:: fill out 1 if any kind of prescription drug is used.; Nulliparity:: fill out 1 if the woman is nulliparous.; Comorbidity:: fill out 1 if any kind of comorbidity is reported.; BMI:: fill out 1 if BMI is between 26 and 30 kg/m^2^.; Number of visits GP:: fill out the number of visits to the GP in the last 10 months. If the woman visited the GP more than three times, fill out 3.

The scores and the predictive probabilities are shown in Table 5. This table can be used by a healthcare provider for decision making during a consult. The mean predictive probability is 0.066 by a score of 0 and 0.574 by a score of 13.

**Table 5:** Scores, predictive probabilities and the percentage of women in each category

**Score**	**Mean predictive probability**	**Sensitivity**	**Specificity**	**Number of persons**	**Number of persons (%)**	**Cumulative percent number of persons**
13	0.574	­	­	1	0.1	100.0
12	-	­	­	-	-	-
11	0.456	­	­	1	0.1	99.9
10	0.411	0.027	0.989	13	1.1	99.8
9	0.349	0.082	0.974	23	2.0	98.7
8	0.301	0.122	0.956	24	2.1	96.7
7	0.258	0.170	0.939	25	2.2	94.6
6	0.217	0.286	0.897	59	5.1	92.5
5	0.182	0.374	0.841	70	6.1	87.4
4	0.155	0.497	0.740	120	10.4	81.3
3	0.124	0.599	0.634	122	10.5	71.0
2	0.102	0.857	0.342	333	28.8	60.4
1	0.081	0.912	0.256	94	8.1	31.6
0	0.066	­	­	272	23.5	23.5

The score is the outcome of the risk score formula:

Risk score = 2 x prescription drug + 2 x nulliparity + 4 x comorbidity + 2 x BMI + number of visits GP.

Prescription drug: fill out 1 if any kind of prescription drug is used.

Nulliparity: fill out 1 if the woman is nulliparous.

Comorbidity: fill out 1 if any kind of comorbidity is reported.

BMI: fill out 1 if BMI is between 26 and 30 kg/m^2^.

Number of visits GP: fill out the number of visits to the GP in the last 10 months. If the woman visited the GP more than three times, fill out 3.

The mean predictive probability is the chance that a women with that score uses OTC-medication. The number of persons are the number of persons in our database having a particular score.

## Discussion

We found that the prevalence of OTC-medication use in a Dutch cohort of pregnant women was 12.5%. Five predictors including nulliparity, prescription drug use, comorbidity, BMI and GP visits could be identified and these predictors were used to design a prediction model which is easy to implement in clinical practice. The prediction model assists healthcare professionals in identifying pregnant women that are likely to use OTC-medication, so information can be given directly to the women at risk.

Although it is difficult to compare because of the divergence in study designs and study populations, the prevalence of 12.5% is low in comparison to similar studies that have been published [4,5,12,13]. An Irish study found a prevalence of 19.5% [13] (2010) and studies from the United States found percentages of women that self-medicated with OTC-medication varying between 92,6% [4] (2003) and 53,6% [5] (1993). A recent study among Hispanic women from the United States found a prevalence of 23% [15] (2010). A Dutch study which was published in 1991 found a prevalence of 45% [16]. Because these data were collected 21 years ago, the population is not representative for the population nowadays. It is likely that pregnant women today are more aware of the risks of medication use during pregnancy and this could explain the low prevalence of OTC-medication use in our study. Another explanation for the large difference in prevalence between our study and the study from 1991 is that de Jong-van den Berg et al. assembled data by an interview of approximately 30 minutes and medication use was ascertained in detail in three different ways. Besides that, all vitamins -except folic acid- were included in their study whereas we excluded vitamin D users. Other studies on drug use during pregnancy performed in the Dutch population are not comparable to our study, because they used data collected from the pharmacy information system or made no distinction between prescribed and self-administered drugs [6,7].

Previous studies described that the use of OTC-medication was positively related to multiple illnesses [4,5], Caucasians [4,5,12], women with more than a high school education [12], women who were at least 20 years of age [12,13], nulliparity [13], smoking [13,14] and being single [13]. We included all these variables in our study, except for marital status, because those data were not available. Besides the five predictors previously mentioned, we also found a non-significant higher frequency of OTC-medication use in non-Western women compared to Western women. Non-Western women were underrepresented (n = 26) in the study due to the exclusion criteria. Therefore, the predictor was not strong enough to include in the multivariate model. We encountered the same power problem for the association between OTC-medication use and the variables alcohol use (n=6), previous prematurity/child mortality (n = 7) and low education (n=7). Therefore, the study results should be interpreted with caution for women with (one of) these characteristics.

Although our results partially differ from the predictors published so far, we were able to design a strong prediction model, with five predictors that independently contribute. Further research with larger cohorts is necessary to study the non-significant associations. The score (Table 5) and the equation (Equation 1) are designed for use in daily practice. Women with a score of 8 or higher have 30.1%-57.4% chance of using OTC-medication during pregnancy. 5.4% of the women included fell into this category. 63% of the women had a score between 2 and 7 and 31.6% of the women had a score between 0 and 1. Explorative analysis showed that the rule performed more or less similar results for subgroups of medications. We suggest the women with a score of 8 or higher, are eligible for comprehensive information provision. The sensitivity and specificity of the model at this cut-off score are 0.122 and 0.956, respectively. This means that the chance of a false positive result is small, while the chance of a false negative result is large. Sensitivities and specificities of the model at other cut-off values are represented in Table 5. The choice of a cut-off score is arbitrary and healthcare providers should make their own considerations concerning the information provision to pregnant women that are at risk for OTC-medication use.

Our study is the first in identifying predictors of OTC-medication use in the Dutch population. Other strong points of our study are the large cohort size (n=1246) and the completeness of the data concerning a variety of socio-demographic, obstetric and medical characteristics. Because our dataset was primarily assembled for a study with another objective and because the questionnaire was filled out prior to delivery, the reporting of OTC-medication use was relatively unbiased. Another strong point of our study is that data were collected by self-reporting. This reflects the reporting of (OTC-)medication use in daily practice.

A limitation of this study is that, for the analyses, OTC-medication use was coded as present or absent and not subdivided per medication group. Explorative analyses of the predictors per subgroup of OTC-medication (e.g. analgesics, vitamins or medication for gastro-intestinal tract) showed that the predictors for each subgroup were comparable to the predictors of overall OTC-medication use (data available on request). However, a larger cohort is necessary to examine the strength of the associations per subgroup.

Another limitation of our study is that the prevalence of OTC-medication use may have been underestimated. The questionnaires have been filled out in different stages of pregnancy, with a range from 13 to 40 weeks. Especially self-reporting of (OTC-)medication later in pregnancy can lead to under-reporting due to poor recall. When a questionnaire is completed earlier in pregnancy the medication use afterwards is not known. Another potential cause of under-reporting is the questionnaire design, because an open-ended question was used to gain information about OTC-medication use. It has been reported that questions involving indication for use and drug-specific questions increase the prevalence estimates compared to open-ended questions. However, healthcare professionals commonly use open-ended questions. Therefore, we think that our data reflect the reporting in daily practice, while the actual use might be under-estimated. Healthcare professionals should take the questionnaire design into account while interpreting the results of this study. A third limitation is that the population is not representative for secondary healthcare. We assume that the predictors we have selected are still relevant in secondary healthcare, but other risk factors may contribute as well. Finally, the participants reported the number of GP visits during pregnancy one-month post-partum. This contradicts with the terms that only predictors that are available at the time the model will be used should be included in the prediction model [11]. Nevertheless we have decided to include this post-partum variable, because it is likely that there is an association between OTC-medication use during pregnancy and the number of GP visits. This association may exist, because pregnant women who visit their GP are more likely to suffer from pregnancy ailments and may use OTC-medication consequently.

A fourth limitation of our study is that the model was not internally validated. Therefore, we may have overestimated the predictive value of the model. Further research is recommended to validate the model and avoid the risk of overestimation.

## Conclusion

Finally, we included five predictors in our model (nulliparity, prescription drug use, comorbidity, BMI26-30 kg/m^2^ and GP visits) and found an area under the ROC-curve of 0.667. This value suggests that our model is moderately discriminating. On the other hand, the variability in predicted probabilities ranged from 6.6% to 57.4% and this indicates that the model is well discriminative. The Hosmer and Lemeshow test validated that the model is statistically fitted (P= 0.640).

To optimize the prediction model there is a need for studies with larger cohorts and studies that ascertain (OTC-)medication use more in detail. However, our study outcome gives a good indication of maternal characteristics that are positively associated with OTC-medication use and provides a risk score that is easy to implement in clinical practice.

## Abbreviations

OTC-medication: Over-the-counter medication; NSAIDs: Non-steroidal anti-inflammatory drugs; DA: Ductus arteriosus; Q fever: Query fever; ATC: Anatomical therapeutic chemical; OR: Odds ratio; CI: Confidence intervals; ROC-curve: Receiver operating characteristic-curve; BMI: Body mass index (kg/m^2^); GP: General Practitioner.

## Competing interests

The authors declare that they have no competing interests

## Authors’ contributions

EJS and GMPJV were responsible for the data analysis, interpretation and drafting the manuscript. JM was responsible for the design and acquisition of data and revising the manuscript. JA revised the manuscript and gave final approval for submission. EH was supervisor of the project, revised the manuscript and gave final approval for submission. All authors read and approved the final manuscript.

## Pre-publication history

The pre-publication history for this paper can be accessed here:

http://www.biomedcentral.com/1471-2458/13/185/prepub
